# Hormone replacement therapy and cancer mortality in women with 17 site-specific cancers: a cohort study using linked medical records

**DOI:** 10.1038/s41416-024-02767-8

**Published:** 2024-06-24

**Authors:** Chris R. Cardwell, Tom A. Ranger, Alexander M. Labeit, Carol A. C. Coupland, Blánaid Hicks, Carmel Hughes, Úna McMenamin, Xue W. Mei, Peter Murchie, Julia Hippisley-Cox

**Affiliations:** 1 Centre for Public Health, Queen’s University Belfast, Belfast, Northern Ireland UK; 2 Nuffield Department of Primary Care Health Sciences, University of Oxford, Oxford, UK; 3 Centre for Academic Primary Care, University of Nottingham, Nottingham, UK; 4 School of Pharmacy, Queen’s University Belfast, Belfast, Northern Ireland UK; 5 Division of Applied Health Sciences Section, Academic Primary Care, University of Aberdeen, Foresterhill, Aberdeen UK

**Keywords:** Cancer epidemiology, Hormonal therapies

## Abstract

**Background:**

There is limited evidence on the safety of Hormone Replacement Therapy (HRT) in women with cancer. Therefore, we systematically examined HRT use and cancer-specific mortality in women with 17 site-specific cancers.

**Methods:**

Women newly diagnosed with 17 site-specific cancers from 1998 to 2019, were identified from general practitioner (GP) records, hospital diagnoses or cancer registries in Scotland, Wales and England. Breast cancer patients were excluded because HRT is contraindicated in breast cancer patients. The primary outcome was time to cancer-specific mortality. Time-dependent Cox regression models were used to calculate adjusted hazard ratios (HR) and 95% confidence intervals (95% CIs) for cancer-specific mortality by systemic HRT use.

**Results:**

The combined cancer cohorts contained 182,589 women across 17 cancer sites. Overall 7% of patients used systemic HRT after their cancer diagnosis. There was no evidence that HRT users, compared with non-users, had higher cancer-specific mortality at any cancer site. In particular, no increase was observed in common cancers including lung (adjusted HR = 0.98 95% CI 0.90, 1.07), colorectal (adjusted HR = 0.79 95% CI 0.70, 0.90), and melanoma (adjusted HR = 0.77 95% CI 0.58, 1.02).

**Conclusions:**

We observed no evidence of increased cancer-specific mortality in women with a range of cancers (excluding breast) receiving HRT.

## Background

Hormone replacement therapy (HRT) is widely used to reduce menopausal related vasomotor symptoms (hot flushes, and night sweats) [1], urogenital atrophy [1] and postmenopausal osteoporosis [2]. It has also been shown to reduce joint pain, mood swings, sleep disturbances and improve quality of life [1]. The earlier detection and improved survival of patients with cancer has led to increasing numbers of women with cancer experiencing menopausal symptoms. In the United Kingdom (UK) HRT is contraindicated in patients with breast cancer and oestrogen-dependent cancers [2–4] but is not contraindicated in patients with other cancers. Clinicians may be reluctant to treat menopausal symptoms in patients with cancer using HRT given uncertainty around the impact of HRT on cancer outcomes, but denial of HRT without clear indication has been criticised by some authors as it could lead to unnecessary suffering [5, 6].

Some researchers summarising evidence on the safety of HRT use in patients with cancer have advised caution when prescribing HRT to patients with several cancers including bladder, gastric and lung cancer [7, 8]. These recommendations were partly based on preclinical studies suggesting that oestrogen stimulates growth in bladder [9] and gastric cancer cell lines [10] and lung cancer mouse models [11]. Further, observational studies have shown increases in the risk of glioma and meningioma with use of oestrogen alone HRT [12] and the Women’s Health Initiative randomised controlled trial (RCT) showed a marked increase in death from lung cancer in the oestrogen plus progestin group compared with placebo [13]. Also, an early cohort study showed reduced survival with prior HRT use in patients with lung cancer [14] but this was not replicated in two later studies [15, 16]. Few epidemiological studies have investigated HRT use after cancer diagnosis and cancer-specific mortality, and to the best of our knowledge none has investigated HRT use after a bladder, gastric or lung cancer diagnosis.

We determined the association between HRT use after cancer diagnosis and the risk of cancer-specific mortality in women with a range of 17 cancers (excluding breast cancer because HRT is contraindicated in patients with breast cancer), to help inform the decision to use HRT in women with cancer.

## Methods

The main methods are described in previously published protocols [17, 18]. The study utilised data sources from QResearch (version 44, England) [19] the Scottish National Prescribing Information System (Scotland) [20] and the SAIL databank (Wales) [21, 22]. QResearch is a general practice database including over 1000 practices and over 10 million patients [19]. QResearch is linked at individual patient level to a range of data sources including hospital admissions data and national mortality records. The Scottish National Prescribing Information System dataset was utilised from 2009 as after this timepoint it is estimated to capture over 95% of prescriptions [20] and was linked to Scottish hospital admissions data which has been shown to be accurate for a wide range of conditions [23]. The SAIL Databank is a repository of health data which allows linkage of data from a number of sources including general practice and hospital admissions data with an accuracy of over 99% [21, 22]. Analyses in Scotland, England and Wales all utilised UK cancer registry data which have high levels of completeness and accuracy over the study period [24]. The study has been reported in accordance with the STROBE guidelines [25].

### Cohorts

Population-based cohorts of women, aged 40–79, newly diagnosed with an incident cancer were identified solely from cancer registry records in Scotland (Scottish Cancer Registry) and Wales (Welsh Cancer Intelligence and Surveillance Unit) and from three data sources in England (general practice (GP) diagnosis codes, hospital diagnoses and cancer registry records from QResearch database). The dates of diagnosis included were: January 1998 to September 2019 in England; January 2009 to December 2016 in Scotland; and January 2000 to December 2016 in Wales. Seventeen of the most common female cancers (excluding breast cancer) were investigated (see Supplementary Table 1 for ICD10 codes used). Patients previously diagnosed with other invasive cancers (apart from non-melanoma skin cancer) were excluded. In a deviation from the published protocols, women with a diagnosis of thyroid cancer were excluded due to small numbers of diagnoses across all three datasets.

### Exposure

HRT use was ascertained from electronic GP prescribing records (Wales and England) or dispensing records (Scotland) which were available from the date of cohort entry. The main HRT definition included systemic oestrogen-containing products (and tibolone) used for menopausal symptoms based upon the British National Formulary [4] classification (Section 6.8.1). Vaginal oestrogen therapy was also identified based upon the British National Formulary classification (contained in Section 7.6.2).

### Outcome

The primary outcome was cancer-specific mortality from national mortality records (based upon the corresponding cancer as the underlying cause of death in England, Scotland and Wales, see Supplementary Table 1 for ICD10 codes used) for each of the 17 cancer sites. Linked national mortality records were available until March 2020 in England, December 2020 in Scotland, and June 2020 in Wales. We investigated the cancer-specific hazard because our primary interest was in the aetiological effect of HRT on cancer-specific mortality in those who were event free. A secondary analysis was conducted on all-cause death which increased power and avoided any potential misclassification of the cause of death.

### Covariates

Cancer treatment (including radiotherapy, chemotherapy and surgery) was determined from cancer registry records in Scotland and Wales and from Hospital Episode Statistics (HES) in England. Cancer stage grouping was determined from cancer registry records except, to minimise missing data, Duke’s stage was used for colorectal cancer in Scotland, Breslow thickness was used for melanoma in Scotland, Figo stage was used for cervical cancer in Scotland and Wales and Figo stage was used for ovarian cancer in Scotland. Charlson comorbidities recorded before cancer diagnosis were identified from GP records and hospital admissions (available from 2000) in Wales, GP records and HES (available from 1998) in England and from hospital admissions alone (available from 1999) in Scotland (apart from diabetes which was identified from diabetes medications in Scotland). Other medication use (including aspirin, statins, metformin and oral contraceptives) was determined at any time before cancer diagnosis from GP prescribing (in England from 1989 and in Wales from 2000) or dispensing records (in Scotland from 2009). Hysterectomy/oophorectomy was determined from hospital admissions data in Scotland and hospital admissions and GP records in Wales and England. Deprivation of home address postcode was determined based upon the relevant 2011 Index of Multiple Deprivation in Scotland and Wales [20, 21] and the 2011 Townsend deprivation score in England [26]. Smoking and BMI was determined from the most recent GP records before cancer diagnosis (not available in Scotland).

### Statistical analysis

In the primary analysis of HRT use after diagnosis, patients were followed from 6 months after cancer diagnosis to cancer-specific mortality and censored on death from other causes, end of follow-up (the latest date at which mortality records were complete) and additionally in England and Wales end of GP records. Consequently, patients who died in the first 6 months after cancer diagnosis were excluded as it seemed unlikely that HRT use after diagnosis could impact these deaths (in sensitivity analyses, this period was increased to 1 year). HRT was modelled as a time varying covariate to avoid immortal time bias [27], i.e. patients were first considered non-users and then users after a lag of 6 months following their first prescribed/dispensed HRT. A lag is recommended in studies of medication use and cancer survival [28]. A time-varying duration-response analysis was conducted with individuals considered a non-user prior to 6 months after first prescribed/dispensed HRT, a short term user from 6 months after first prescribed/dispensed HRT to 6 months after their fifth prescribed/dispensed HRT, and a longer term user after this time. The fifth prescribed/dispensed HRT was used because in a preliminary analysis this roughly corresponded to 1 year of HRT prescriptions. Time-dependent Cox regression models were used to calculate hazard ratios (HRs), and 95% confidence intervals (CIs), comparing users of HRT with non-users after cancer diagnosis adjusting for age at diagnosis, year of diagnosis, deprivation, Charlson comorbidity (before diagnosis), anaemia (before diagnosis) other medication use (including statins, aspirin, metformin and oral contraceptives before diagnosis), cancer treatment (including surgery, chemotherapy and radiotherapy), and hysterectomy/oophorectomy (in the periods up to 1 month before cancer diagnosis and 1 month before to 6 months after cancer diagnosis). The Cox proportional hazards assumption was checked by visual inspection of log(−log) plots and appeared to be largely satisfied. Estimates were calculated within each cohort and then pooled using random effects meta-analysis models [29].

Analyses were repeated for all-cause mortality. An additional analysis was conducted at all sites, additionally adjusting for BMI (at diagnosis, based upon complete case), from GP records before diagnosis, restricted to England and Wales. Additional analyses were conducted for the following more common cancers: colorectal, melanoma, ovarian, lung and cervical cancer. Analyses were conducted with type of HRT coded as a single time-varying variable, with a lag of 6 months, into the following hierarchical categories: combined HRT (with or without other HRT), oestrogen-only HRT (with or without tibolone) and tibolone only. HRT type was compared with no HRT use. A number of sensitivity analyses were conducted. First, the lag was increased to 1 year (with follow-up starting at one year after cancer diagnosis). Second, analyses were repeated with vaginal oestrogen therapy (mainly oestradiol pessaries and oestriol creams) included within the HRT definition. Third, systemic users of HRT were compared with users of vaginal oestrogen therapy, as users of vaginal oestrogen therapy are likely to share indications but receive much lower amounts of oestrogen than systemic users. In this analysis HRT was included using a single time-varying covariate (lagged by 6 months) coded as systemic HRT use (with or without vaginal oestrogen therapy use), vaginal oestrogen therapy use and no HRT use. Fourth, analyses was conducted restricting to stage 1 to 3 disease and restricting to stage 1 and 2 disease. Fifth, analyses were conducted varying the age range: restricting to women aged over 55 (who are more likely to be post-menopausal and widening to women aged 18–79 years. Sixth, a new user analysis was conducted restricted to women who had not used HRT in the period 18 to 6 months before diagnosis. Seventh, an analysis was conducted adjusting for cancer stage using multiple imputation. Stage was imputed in 10 imputed datasets using ordinal logistic regression models with cancer-specific death status, cumulative hazard along with all confounders from the adjusted model included in imputation models [30], and results were combined using Rubin’s rules [31]. Eighth, analyses were conducted additionally adjusting for cancer stage (based upon complete case) and additionally adjusting for cancer stage and smoking (based upon complete case, restricted to England and Wales). Finally, an analysis was conducted for death from cardiovascular disease (based upon ICD10 codes I20 to I99 or G45 as the underlying cause of death).

A separate analysis was conducted to investigate HRT use before diagnosis. In this analysis patients were followed from the date of cancer diagnosis to cancer-specific mortality (censored as previously) and HRT use was defined as one or more prescribed/dispensed systemic HRT in the period 18 months to 6 months before diagnosis. HRT use in the 6 months immediately before diagnosis was ignored because medication use can increase in this period due to cancer symptoms [32] which may be more marked in patients with advanced cancer. HRs (and 95% CIs) were calculated using Cox regression adjusting for age, year, deprivation, Charlson comorbidity (before diagnosis), other medication use (before diagnosis) and hysterectomy/oophorectomy (up to 1 month before cancer diagnosis). STATA 16/17 was used for all analyses. Analysis code is available from the authors upon request.

### Ethical approval

Ethical approval for English data was obtained from the QResearch scientific committee (Ref: OX24, project title ‘Use of hormone replacement therapy and survival from cancer’). Ethical approval for the QResearch database is obtained annually from East Midlands - Derby Research Ethics Committee (Ref:18/EM/0400). Approval for analysis of the Welsh data has been obtained from the SAIL Databank Information Governance Review Panel (Reference: 0965) and approval for the analysis of the Scottish data has been obtained from the Public Benefit and Privacy Panel for Health and Social Care (Reference: 2021-0014).

## Results

The final cohort contained 182,589 patients with cancer, who survived more than 6 months after their cancer diagnosis, across 17 cancer sites followed for 840,133 person years. There were 54,861 cancer-specific deaths during follow-up. Overall, 7% (11,972) of patients with cancer used systemic HRT after cancer diagnosis. For instance, 5% of patients with colorectal cancer, 4% of patients with lung cancer and 11% of patients with malignant melanoma used systemic HRT after cancer diagnosis.

### Characteristic of users and non-users of HRT

The characteristics of users and non-users of HRT are shown in Table 1 and Supplementary Table 2. In general, users of HRT, compared with non-users, were younger at diagnosis, had higher rates of hysterectomy/oopherectomy, and smoking. Also, a lower proportion of users of HRT had diabetes or chronic kidney disease and had been prescribed statins, aspirin or metformin and a higher proportion had been prescribed oral contraceptives. A lower proportion of users of HRT received chemotherapy than HRT non-users. The distribution of stage was fairly similar for women with colorectal and lung cancer in users of HRT compared with HRT non-users, but a greater proportion of HRT users had stage 1 and 2 disease for women with cervical and ovarian cancer. Other characteristics of users of HRT and non-users were largely similar.

**Table 1:** Patient characteristics by hormone replacement therapy use after cancer diagnosis in women with at least 6 months of follow-up.

	England		Scotland		Wales	
	HRT non-user	HRT user	HRT non-user	HRT user	HRT non-user	HRT user
	(*n* = 98884)	(*n* = 8022)	(*n* = 44711)	(*n* = 2141)	(*n* = 27022)	(*n* = 1809)
Age						
40–49	8786 (9%)	2545 (32%)	3724 (8%)	957 (45%)	2201 (8%)	692 (38%)
50–59	19449 (20%)	2955 (37%)	9272 (21%)	765 (36%)	5746 (21%)	636 (35%)
60–69	32954 (33%)	1815 (23%)	15315 (34%)	321 (15%)	9484 (35%)	366 (20%)
70–79	37695 (38%)	707 (9%)	16400 (37%)	98 (5%)	9591 (35%)	115 (6%)
Year of diagnosis						
1998–2004	17916 (18%)	3135 (39%)			6160 (23%)	722 (40%)
2005–2009	22722 (23%)	1737 (22%)	4241 (9%)	227 (11%)	7780 (29%)	478 (26%)
2010–2014	27676 (28%)	1639 (20%)	22168 (50%)	1098 (51%)	9182 (34%)	444 (25%)
2015–2019	30570 (31%)	1511 (19%)	18302 (41%)	816 (38%)	3900 (14%)	165 (9%)
Deprivation						
1st fifth (most deprived)	30373 (31%)	2655 (33%)	9831 (22%)	442 (21%)	5360 (20%)	328 (18%)
2nd fifth	24651 (25%)	2090 (26%)	9412 (21%)	378 (18%)	5096 (19%)	324 (18%)
3rd fifth	19170 (19%)	1521 (19%)	8790 (20%)	456 (21%)	5564 (21%)	366 (20%)
4th fifth	14155 (14%)	1055 (13%)	8699 (19%)	483 (23%)	4769 (18%)	341 (19%)\
5th fifth (least deprived)	10414 (11%)	688 (9%)	7947 (18%)	377-382^b^ (%)	5479 (20%)	398 (22%)
Missing	121 (0%)	13 (0%)	32 (0%)	0–5 ^b^ (%)	754 (3%)	52 (3%)
Smoking before diagnosis						
Never	51006 (52%)	3829 (48%)			11637 (43%)	600 (33%)
Past	18677 (19%)	1491 (19%)			5785 (21%)	282 (16%)
Current	24101 (24%)	2337 (29%)			5241 (19%)	449 (25%)
Missing	5100 (5%)	365 (5%)			4359 (16%)	478 (26%)
Hysterectomy / oophorectomy^a^						
Before cancer	15741 (16%)	2145 (27%)	1399 (3%)	183 (9%)	1663 (6%)	193 (11%)
At cancer diagnosis	18261 (19%)	1800 (22%)	7456 (17%)	481 (23%)	6105 (23%)	492 (27%)
After cancer diagnosis	1896 (2%)	349 (4%)	827 (2%)	84 (4%)	552 (2%)	102 (6%)
BMI (kg/m^2^): mean (sd)^c^	27.7 (6.0)	26.2 (5.2)			28.7 (6.9)	27.1 (5.9)
Comorbidity (any time before diagnosis)						
Myocardial infarction	2226 (2%)	65 (1%)	1521 (3%)	20 (1%)	743 (3%)	16 (1%)
Congestive heart failure	1638 (2%)	35 (0%)	882 (2%)	12 (1%)	735 (3%)	17 (1%)
Peripheral vascular disease	1788 (2%)	57 (1%)	1096 (2%)	13 (1%)	872 (3%)	45 (2%)
Stroke	4268 (4%)	121 (2%)	974 (2%)	13 (1%)	1011 (4%)	17 (1%)
COPD	6839 (7%)	308 (4%)	3164 (7%)	61 (3%)	2479 (9%)	82 (5%)
Hemiplegia	440 (0%)	7 (0%)	256 (1%)	8 (0%)	253 (1%)	11 (1%)
Dementia	1036 (1%)	35 (0%)	155 (0%)	0–5 ^b^ (%)	194 (1%)	0–5 ^b^ (%)
Liver diseases	4108 (4%)	254 (3%)	967 (2%)	19 (1%)	575 (2%)	30 (2%)
Peptic ulcer	10779 (11%)	313 (4%)	808 (2%)	31 (1%)	798 (3%)	32 (2%)
Diabetes	6494 (7%)	133 (2%)	4073 (9%)	63 (3%)	3249 (12%)	92 (5%)
Chronic kidney disease	12906 (13%)	746 (9%)	943 (2%)	13 (1%)	2132 (8%)	47 (3%)
Anaemia	2226 (2%)	65 (1%)	1652 (4%)	33 (2%)	3064 (11%)	139 (8%)
Medication use (any time before diagnosis)						
Statin	29263 (30%)	928 (12%)	15640 (35%)	287 (13%)	8618 (32%)	238 (13%)
Aspirin	22343 (23%)	831 (10%)	9987 (22%)	182 (9%)	6196 (23%)	168 (9%)
Metformin	8220 (8%)	231 (3%)	3331 (7%)	49 (2%)	2102 (8%)	57 (3%)
Oral contraceptive	8612 (9%)	1592 (20%)	1116 (2%)	238 (11%)	1046 (4%)	237 (13%)
Cancer treatment						
Surgery	48537 (49%)	4191 (52%)	26739 (60%)	1522 (71%)	21232 (79%)	1419 (78%)
Chemotherapy	29753 (30%)	1757 (22%)	18533 (41%)	773 (36%)	8033 (30%)	476 (26%)
Radiotherapy	7355 (7%)	397 (5%)	10528 (24%)	433 (20%)	1753 (6%)	78 (4%)

^a^Hysterectomy/oophorectomy in the following time periods: before cancer (up to 1 month before cancer diagnosis), at cancer diagnosis (from 1 month before cancer diagnosis to 6 months after cancer diagnosis) and after cancer diagnosis (more than 6 months after cancer diagnosis).

^b^Range shown to maintain statistical disclosure control.

^c^BMI available for 84,285 HRT non- users and 6685 HRT users in England and 19,161 HRT non-users and 1097 HRT users in Wales.

### HRT use after cancer diagnosis and cancer-specific mortality

**Fig. 1:**
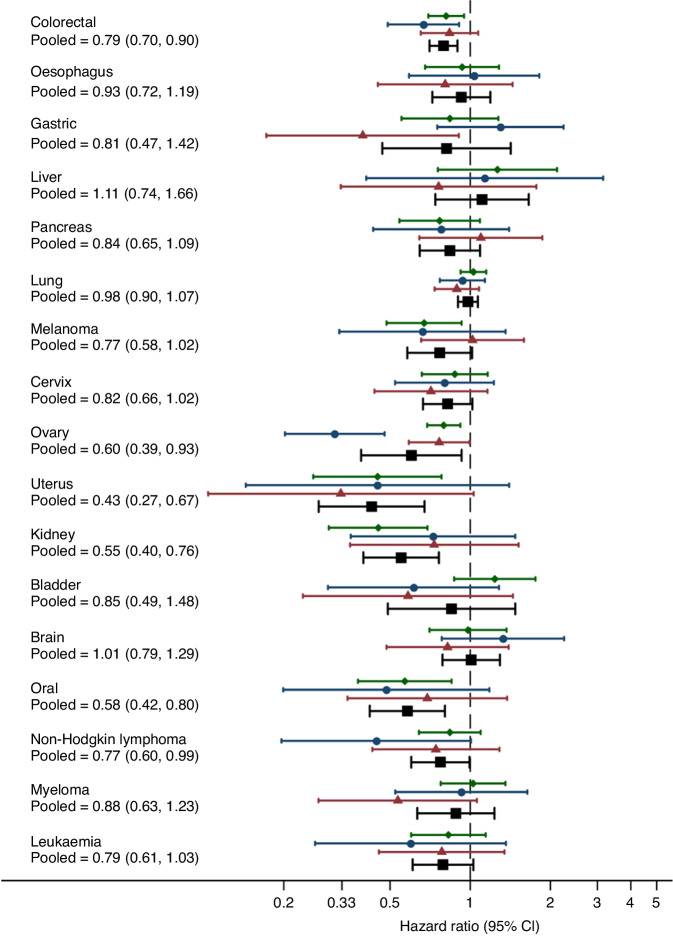
Adjusted hazard ratios for the association between hormone replacement therapy use after diagnosis and cancer-specific mortality in England (), Scotland (), Wales () and pooled (), by site.

The pooled associations between HRT use after diagnosis and cancer-specific mortality are shown in Table 2 and Fig. 1. There was no evidence of higher cancer-specific mortality in users of HRT after diagnosis, compared with non-users, at any of the 17 cancer sites studied. In contrast, use of HRT compared with non-use was associated with a lower rate of cancer-specific mortality for colorectal cancer (adjusted HR = 0.79 95% CI 0.70, 0.90), ovarian cancer (adjusted HR = 0.60 95% CI 0.39, 0.93), uterus cancer (adjusted HR = 0.43 95% CI 0.27, 0.67), kidney cancer (adjusted HR = 0.55 95% CI 0.40, 0.76), oral cancer (adjusted HR = 0.58 95% CI 0.42, 0.80) and non-Hodgkin lymphoma (adjusted HR = 0.77 95% CI 0.60, 0.99). However, the analysis of uterus and kidney was based upon relatively small numbers of cancer-specific deaths in users of HRT (less than 40) and in further analysis of oral cancer and non-Hodgkin lymphoma the association did not follow a dose-response as there was no association in patients with 5 or more prescriptions compared with no prescriptions (adjusted HR = 0.87 95% CI 0.57, 1.32 and adjusted HR = 0.89 95% CI 0.65, 1.21, respectively). Associations were generally similar in analyses additionally adjusting for BMI (in England and Wales) shown in Supplementary Table 3.

**Table 2:** Pooled analyses of hormone replacement therapy use after diagnosis and cancer-specific mortality in England, Scotland and Wales.

Cancer site	HRT user		HRT non-user		HRT user v non-user			1 to 4 prescriptions		5 or more prescriptions	
	Cancer- deaths	Person-years	Cancer- deaths	Person-years	Unadjusted HR (95% CI)	Adjusted^a^ HR (95% CI)	*P*^b^	Adjusted^a^ HR (95% CI)	P^b^	Adjusted^a^ HR (95% CI)	*P*^b^
Colorectal	289	11256	8192	167432	0.80 (0.70, 0.91)	0.79 (0.70, 0.90)	<0.001	0.79 (0.68, 0.92)	0.002	0.80 (0.67, 0.97)	0.023
Oesophagus	67	568	2367	8818	0.84 (0.66, 1.07)	0.93 (0.72, 1.19)	0.547	0.96 (0.72, 1.27)	0.776	0.90 (0.50, 1.62)	0.728
Gastric	44	803	1664	10758	0.70 (0.45, 1.10)	0.81 (0.47, 1.42)	0.469	0.90 (0.57, 1.43)	0.667	0.75 (0.35, 1.62)	0.465
Liver	23-28^d^	103	1051	3612	1.09 (0.74, 1.60)	1.11 (0.74, 1.66)	0.622	1.18 (0.74, 1.87)	0.482	0.93 (0.43, 2.02)	0.857
Pancreas	61	337	2764	6317	0.75 (0.57, 0.97)	0.84 (0.65, 1.09)	0.186	0.90 (0.59, 1.38)	0.632	0.82 (0.47, 1.43)	0.482
Lung	573	2986	15931	55229	0.97 (0.87, 1.08)	0.98 (0.90, 1.07)	0.648	0.95 (0.81, 1.12)	0.557	0.99 (0.82, 1.21)	0.958
Melanoma	74	12877	1078	95824	0.68 (0.50, 0.93)	0.77 (0.58, 1.02)	0.065	0.96 (0.70, 1.30)	0.779	0.59 (0.34, 1.05)	0.071
Cervix	111	7862	1102	22788	0.46 (0.34, 0.62)	0.82 (0.66, 1.02)	0.073	0.93 (0.71, 1.21)	0.571	0.71 (0.52, 0.97)	0.031
Ovary	321	10350	5735	58518	0.39 (0.27, 0.57)	0.60 (0.39, 0.93)	0.022	0.76 (0.53, 1.09)	0.142	0.47 (0.26, 0.85)	0.012
Uterus	13-23 ^d^	5837	2375	124788	0.27 (0.17, 0.42)	0.43 (0.27, 0.67)	<0.001	0.45 (0.24, 0.83)	0.011	0.54 (0.28, 1.04)	0.066
Kidney	39	2848	1507	34047	0.48 (0.35, 0.66)	0.55 (0.40, 0.76)	<0.001	0.55 (0.36, 0.84)	0.005	0.59 (0.34, 1.04)	0.07
Bladder	43-48 ^d^	3100	1395	38680	0.79 (0.59, 1.06)	0.85 (0.49, 1.48)	0.565	0.95 (0.51, 1.75)	0.866	0.83 (0.49, 1.43)	0.506
Brain	70	473	1629	5621	0.94 (0.74, 1.19)	1.01 (0.79, 1.29)	0.947	1.01 (0.76, 1.35)	0.931	1.01 (0.64, 1.57)	0.982
Oral	36-41 ^d^	2614	1172	28711	0.60 (0.44, 0.83)	0.58 (0.42, 0.80)	0.001	0.41 (0.25, 0.68)	0.001	0.87 (0.57, 1.32)	0.502
NHL^c^	83	5293	1986	56960	0.58 (0.41, 0.81)	0.77 (0.60, 0.99)	0.044	0.70 (0.51, 0.97)	0.031	0.89 (0.65, 1.21)	0.443
Myeloma	81	1322	1490	18029	0.76 (0.55, 1.04)	0.88 (0.63, 1.23)	0.466	0.75 (0.53, 1.05)	0.097	0.89 (0.41, 1.95)	0.776
Leukaemia	63	3011	1412	32361	0.68 (0.53, 0.88)	0.79 (0.61, 1.03)	0.079	0.83 (0.59, 1.18)	0.297	0.77 (0.53, 1.13)	0.182

^a^Adjusted model contains age, year of diagnosis, deprivation, cancer treatment (surgery, radiotherapy, chemotherapy), Charlson comorbidities (before diagnosis), anaemia (before diagnosis), medication use (before diagnosis: statin, aspirin, metformin and oral contraceptive) and hysterectomy/oophorectomy (before or at diagnosis).

^b^*P* value from adjusted Cox regression model.

^c^Non-Hodgkin lymphoma.

^d^Range shown to maintain statistical disclosure control.

Additional analyses conducted for colorectal, lung, melanoma, cervical and ovarian cancer are shown in Table 3 and Supplementary Table 4. The inverse association between HRT and cancer-specific mortality in patients with colorectal cancer was similar in most sensitivity analyses. However, the association was attenuated when systemic users of HRT were compared with users of vaginal oestrogen therapy (adjusted HR = 0.93 95% CI 0.78, 1.10) and in the analysis restricted to stage 1–3 patients with colorectal cancer (adjusted HR = 0.87 95% CI 0.68, 1.11). The null association between HRT and cancer-specific mortality in patients with lung cancer (adjusted HR = 0.98 95% CI 0.90, 1.07) was generally consistent across sensitivity analyses, except for the analysis comparing systemic to vaginal oestrogen therapy users in which a slight increase was observed (adjusted HR = 1.18 95% CI 1.01, 1.39). The null association between HRT and cancer-specific mortality in patients with melanoma and cervical cancer was also fairly similar across sensitivity analyses. The inverse association between HRT use and cancer-specific mortality in patients with ovarian cancer was similar in most sensitivity analyses. Further, analyses by HRT type are shown in Supplementary Table 5. There were no marked differences in associations by HRT type for patients with colorectal cancer, lung cancer or melanoma. In patients with cervical cancer and ovarian cancer an inverse association was observed solely in patients on oestrogen-only HRT compared with no HRT use (adjusted HR = 0.65 95% CI 0.47, 0.91 and adjusted HR = 0.55 95% CI 0.38, 0.79, respectively).

**Table 3:** Pooled sensitivity analyses of hormone replacement therapy after diagnosis and cancer-specific mortality in England, Scotland and Wales.

Analysis	Cancer- deaths	Person-years	Unadjusted HR (95% CI)	Adjusted^e^ HR (95% CI)
Colorectal				
Main analysis^a^	8481	178687	0.80 (0.70, 0.91)	0.79 (0.70, 0.90)
Using 1 year lag	6451	162372	0.85 (0.75, 0.97)	0.83 (0.73, 0.95)
HRT versus vaginal oestrogen therapy^b^	528	22942	0.98 (0.82, 1.17)	0.93 (0.78, 1.10)
Restricted to age 55 to 79	7216	151411	0.79 (0.68, 0.92)	0.82 (0.70, 0.95)
Restricted to new HRT user^c^	7642	157327	0.85 (0.70, 1.02)	0.83 (0.69, 0.99)
Restricted to stage 1 to 3^d^	3959	128031	0.84 (0.59, 1.19)	0.87 (0.68, 1.11)
Adjusted for stage (MI)^e^	8481	178687	0.80 (0.70, 0.91)	0.82 (0.72, 0.92)
Adjusted for stage (CC)^f^	6868	143268	0.83 (0.68, 1.02)	0.77 (0.63, 0.94)
Lung				
Main analysis^a^	16504	58215	0.97 (0.87, 1.08)	0.98 (0.90, 1.07)
Using 1 year lag	9540	46988	0.97 (0.86, 1.09)	0.99 (0.88, 1.10)
HRT versus vaginal oestrogen therapy^b^	806	5267	1.29 (1.10, 1.51)	1.18 (1.01, 1.39)
Restricted to age 55 to 79	14839	51181	0.98 (0.89, 1.08)	0.99 (0.90, 1.09)
Restricted to new HRT user^c^	14638	51437	0.88 (0.60, 1.29)	0.89 (0.72, 1.10)
Restricted to stage 1 to 3^d^	5653	30872	0.90 (0.64, 1.27)	1.09 (0.85, 1.39)
Adjusted for stage (MI)^e^	16504	58215	0.97 (0.87, 1.08)	0.99 (0.88, 1.12)
Adjusted for stage (CC)^f^	11886	41971	1.01 (0.86, 1.19)	1.08 (0.88, 1.32)
Melanoma				
Main analysis^a^	1152	108702	0.68 (0.50, 0.93)	0.77 (0.58, 1.02)
Using 1 year lag	995	100300	0.63 (0.45, 0.87)	0.69 (0.51, 0.92)
HRT versus vaginal oestrogen therapy^b^	115	20604	1.03 (0.53, 1.99)	1.22 (0.69, 2.18)
Restricted to age 55 to 79	840	66599	0.75 (0.37, 1.52)	0.88 (0.48, 1.63)
Restricted to new HRT user^c^	1009	93485	0.78 (0.55, 1.12)	0.97 (0.68, 1.40)
Restricted to stage 1 to 3^d^	421	49897	0.73 (0.47, 1.14)	0.96 (0.60, 1.52)
Adjusted for stage (MI)^e^	1152	108702	0.68 (0.50, 0.93)	0.85 (0.66, 1.11)
Adjusted for stage (CC)^f^	543	51361	0.64 (0.41, 0.99)	0.89 (0.59, 1.36)
Cervix				
Main analysis^a^	1213	30649	0.46 (0.34, 0.62)	0.82 (0.66, 1.02)
Using 1 year lag	906	28011	0.47 (0.36, 0.61)	0.79 (0.62, 1.01)
HRT versus vaginal oestrogen therapy^b^	134	9321	0.70 (0.27, 1.84)	1.11 (0.54, 2.27)
Restricted to age 55 to 79	714	11423	0.39 (0.21, 0.74)	0.59 (0.31, 1.11)
Restricted to new HRT user^c^	1083	27043	0.50 (0.37, 0.68)	0.90 (0.72, 1.14)
Restricted to stage 1 to 3^d^	454	14126	0.49 (0.32, 0.76)	0.91 (0.65, 1.28)
Adjusted for stage (MI)^e^	1213	30649	0.46 (0.34, 0.62)	0.98 (0.78, 1.24)
Adjusted for stage (CC)^f^	940	21017	0.48 (0.33, 0.69)	0.98 (0.73, 1.32)
Ovary				
Main analysis^a^	6056	68868	0.39 (0.27, 0.57)	0.60 (0.39, 0.93)
Using 1 year lag	4923	61547	0.38 (0.25, 0.58)	0.59 (0.36, 0.95)
HRT versus vaginal oestrogen therapy^b^	439	13300	0.55 (0.31, 0.96)	0.74 (0.41, 1.33)
Restricted to age 55 to 79	4936	44682	0.86 (0.70, 1.04)	0.88 (0.70, 1.11)
Restricted to new HRT user^c^	5324	60248	0.29 (0.20, 0.42)	0.56 (0.36, 0.86)
Restricted to stage 1 to 3^d^	2627	34050	0.37 (0.21, 0.66)	0.77 (0.63, 0.94)
Adjusted for stage (MI)^e^	6056	68868	0.39 (0.27, 0.57)	0.75 (0.58, 0.97)
Adjusted for stage (CC)^f^	4216	41807	0.38 (0.23, 0.65)	0.75 (0.58, 0.98)

^a^Adjusted model contains age, year of diagnosis, deprivation, cancer treatment (surgery, radiotherapy, chemotherapy), Charlson comorbidities (before diagnosis), anaemia (before diagnosis), medication use (before diagnosis: statin, aspirin, metformin and oral contraceptive) and hysterectomy/oophorectomy (before or at diagnosis).

^b^Individuals not using HRT or vaginal oestrogen therapy excluded.

^c^Restricted to patients not using systemic HRT in the period 18 to 6 months before cancer diagnosis.

^d^Restricted to patients stage 1–3. Adjusted model contains all terms in ^a^ along with stage.

^e^Stage imputed using multiple imputation as described in methods. Adjusted model contains all terms in ^a^ along with stage.

^f^Restricted to patients with available stage. Adjusting for stage using complete case, model contains all terms in ^a^ along with stage.

### HRT use after cancer diagnosis and all-cause mortality

Analysis of the association between HRT use after diagnosis and all-cause mortality, shown in Supplementary Table 6, did not reveal any evidence of an increase in all-cause mortality in patients with cancer using HRT at any of the 17 sites studied.

### HRT use before diagnosis and cancer-specific mortality

The pooled association between HRT use before diagnosis and cancer-specific mortality is shown in Table 4 and Supplementary Figure 1. There was no evidence that HRT before diagnosis was associated with higher cancer-specific mortality in patients with cancer at any of the 17 sites studied. There were inverse associations between HRT use, compared with HRT non-use, before diagnosis and cancer-specific mortality for patients with colorectal cancer (adjusted HR = 0.86 95% CI 0.77, 0.97), cervical cancer (adjusted HR = 0.69 95% CI 0.49, 0.98), oral cancer (adjusted HR = 0.72 95% CI 0.55, 0.95) and non-Hodgkin’s lymphoma (adjusted HR = 0.79, 0.66, 0.95).

**Table 4:** Pooled analysis of hormone replacement therapy use before diagnosis and cancer-specific mortality in England, Scotland and Wales.

Cancer site	HRT user		HRT non-user		HRT user v non-user			
	Cancer- deaths	Person-years	Cancer- deaths	Person-years	Unadjusted HR (95% CI)	P^b^	Adjusted^a^ HR (95% CI)	P^c^
Colorectal	442	10188	10363	156337	0.82 (0.71, 0.93)	0.003	0.86 (0.77, 0.97)	0.013
Oesophagus	136	615	3366	9359	0.81 (0.69, 0.97)	0.019	0.93 (0.78, 1.11)	0.405
Gastric	121	816	2798	11012	0.85 (0.71, 1.02)	0.085	0.91 (0.75, 1.09)	0.307
Liver	82	233	2227	4177	0.81 (0.65, 1.01)	0.06	0.87 (0.69, 1.09)	0.212
Pancreas	302	564	6376	8062	0.82 (0.73, 0.92)	0.001	0.88 (0.73, 1.06)	0.178
Lung	1519	3946	29327	62587	0.90 (0.82, 0.98)	0.015	0.95 (0.88, 1.02)	0.145
Melanoma	83	9409	1051	90680	0.86 (0.69, 1.08)	0.201	0.86 (0.68, 1.08)	0.197
Cervix	30-35	1313	1354	26350	0.64 (0.46, 0.90)	0.011	0.69 (0.49, 0.98)	0.037
Ovary	496	5363	6761	60621	0.85 (0.64, 1.12)	0.249	0.97 (0.85, 1.11)	0.673
Uterus	76	5714	2548	116211	0.77 (0.56, 1.06)	0.111	1.00 (0.74, 1.33)	0.977
Kidney	101	2448	2197	32859	0.75 (0.61, 0.92)	0.006	0.84 (0.68, 1.03)	0.095
Bladder	73	2850	1930	35993	0.71 (0.56, 0.89)	0.003	0.82 (0.65, 1.04)	0.101
Brain	190	507	2867	6420	0.86 (0.74, 1.00)	0.046	0.91 (0.78, 1.06)	0.211
Oral	68	2366	1378	27322	0.73 (0.57, 0.93)	0.011	0.72 (0.55, 0.95)	0.021
Non-Hodgkin lymphoma	122	4356	2612	53874	0.67 (0.55, 0.81)	<0.001	0.79 (0.66, 0.95)	0.014
Myeloma	101	1220	1597	17712	0.83 (0.53, 1.32)	0.438	1.02 (0.72, 1.45)	0.9
Leukaemia	97	2320	2146	30422	0.63 (0.42, 0.95)	0.026	0.77 (0.57, 1.05)	0.1

^a^Adjusted model contains age, year, deprivation, Charlson comorbidities (before diagnosis), medication use (before diagnosis: statin, aspirin, metformin, oral contraceptive) and hysterectomy/oophorectomy (before diagnosis).

^b^*P* value from unadjusted Cox regression model.

^c^*P* value from adjusted Cox regression model.

## Discussion

Overall, there was no evidence that patients with any of the 17 cancers studied who took HRT following their cancer diagnosis had higher rates of cancer-specific or all-cause mortality. Use of HRT was associated with reductions in cancer-specific mortality in women with colorectal, ovarian, uterus, kidney, oral and non-Hodgkin lymphoma, but these associations were based upon relatively small numbers or were generally not consistent across sensitivity analyses.

There has been limited previous research on the safety of HRT use after diagnosis in cancer patients. Small randomised controlled trials have been conducted investigating HRT use and survival in patients with ovarian cancer (showing reduced mortality but not progression free survival) [33], endometrial cancer (showing no association) [34] and breast cancer [35], but not at other cancer sites. Observational studies have investigated HRT and survival for patients with ovarian, endometrial, colorectal, melanoma and lung cancer. These observational studies showed a reduced risk of mortality in users of HRT with ovarian cancer [36], a reduced cancer recurrence in users of HRT with endometrial cancer [37] and a reduced cancer-specific mortality with current use of HRT in patients with colorectal cancer [38]. Mixed associations were observed between HRT and survival in previous smaller studies of patients with melanoma [39, 40] and lung cancer [14–16]. To our knowledge, observational studies have not been conducted investigating HRT use after diagnosis and survival for the other cancers sites studied.

Previous reviews of the oncologic safety of HRT have recommended, based upon preclinical and other evidence, that patients with bladder [7], lung [7, 8], brain [7] and gastric cancer [7] avoid HRT. Our study does not provide evidence of increased cancer-specific mortality in users of HRT with bladder, brain or gastric cancer. In most analyses of lung cancer there was no evidence of association but in a sensitivity analysis comparing users of systemic HRT with users of vaginal oestrogen therapy, there was a slight association with increased cancer-specific mortality. Consequently, further research on HRT use in patients with lung cancer is merited.

Our study has several strengths and limitations. The study utilised data from three independent population-based data sources containing over 180,000 patients with cancer with follow-up of up to 21 years. The safety of HRT after diagnosis has not been previously investigated at many of the cancer sites studied. The use of prescribing/dispensing records will have eliminated recall bias and should capture all HRT use because, at the time of the study, HRT was only available by prescription in the UK. However, these data sources do not contain information on actual adherence to HRT.

The main weakness of our study is that HRT was not randomly allocated and hence HRT users may differ from HRT non-users in ways which influence cancer-specific mortality resulting in confounding. We accounted for potential confounding by adjusting for a wide range of confounders, but we cannot rule out residual confounding from unavailable variables (such as parity, age at menopause or alcohol consumption) or incomplete variables (such as smoking). Further, we did not have comprehensive information on contraindications for HRT (e.g. abnormal liver functions tests) and could not reliably adjust for these. Also, across the three countries there will be differential capture of some confounders due to the sources used (for instance, rates of cancer treatments were different between countries). It is possible that users of HRT have healthier lifestyles in general [41] and may have lower body mass index and be more physically active both of which have been shown to be associated with better cancer survival [42, 43]. It is also possible that users of HRT will have more contact with their GP and be more likely to attend screening and other diagnostic examinations also leading to improved outcomes. Family history of cancer may also impact on the decision to use HRT and cancer-specific mortality. Patients with cancer who have a better prognosis may be more likely to receive HRT, because such patients may be more concerned about their quality of life and clinicians may be more inclined to prescribe HRT to them, leading to artificially better survival in patients on HRT. There was no association with increased cancer-specific mortality when investigating HRT before diagnosis (in which this latter bias would not occur). In cancer sites where stage was available, there was no evidence of an association of increased cancer specific mortality with HRT use after diagnosis following adjustment for stage, and a range of potential confounders. We cannot rule out residual confounding by stage for cancer sites where stage was not complete or confounding by stage for cancer sites where stage was not available. The analyses based upon the multiple imputation of stage relies upon the assumption that stage data are missing at random, which we cannot test, and which could be violated if patients with missing data were more likely to have worse stage even after adjusting for variables in the imputation models. We conducted an active comparator analysis comparing systemic with vaginal oestrogen therapy users (who are likely to share many indications and risk factors but will have markedly lower exposure to oestrogen) to attempt to reduce confounding by indication [44]. In our analysis some women using combined HRT consisting of a prescription for oestrogen-only HRT and a separately prescribed progestogen (such as the levonorgestrel-releasing intrauterine system) may have been misclassified as oestrogen-only users of HRT. There remains the possibility of Type 2 error particularly at rarer cancer sites and sites with limited HRT use.

Importantly, there is a particular risk of confounding by indication in women with oestrogen-sensitive cancers such as uterus, ovarian and cervical cancer. This bias could incorrectly lead to null or even inverse associations if women perceived to be at lower risk of recurrence are more likely to receive HRT. Consequently our findings for these cancer sites should be interpreted particularly cautiously. Also, we could not investigate rarer or specific subtypes of certain cancers which may be particularly oestrogen-sensitive, because of small numbers and/or lack of data, and our results cannot be extrapolated to these groups; for instance oestrogen receptor positive gastric [7], oestrogen receptor positive bladder cancer [7], endometrial stromal sarcoma [45], granulosa cell ovarian cancer [45], low-grade serous ovarian [45] and cervical adenocarcinoma [45].

Many limitations of our study reflect the use of routinely collected data which does not contain sufficient detail on tumour type or covariates. There is a need for prospective cohort studies which can capture detailed information on specific potential confounders such as family history, physical activity and alcohol intake as well as ensuring comprehensive data on tumour characteristics including histological classification and stage.

Our study may provide some reassurance to clinicians and patients of the safety of systemic HRT in women with one of the 17 cancers studied, but as stated above should be interpreted cautiously in women with oestrogen-sensitive cancers. Along with other known risks associated with HRT use [46, 47], our findings may contribute to the decision of cancer patients, and their prescribers, to use HRT.

In conclusion, in this large observational study we observed little consistent evidence of an association between HRT use and increased cancer-specific mortality in women with any of the 17 cancers studied.

### Supplementary information

Supplementary Information

## Data Availability

The datasets from Scotland (eDRIS; Public Health Scotland, https://www.isdscotland.org/products-and-services/edris/), Wales (SAIL databank; Swansea University, https://saildatabank.com/) and England (QResearch; University of Oxford, https://www.qresearch.org/) were obtained under strict data access conditions that allowed the study to be conducted but do not allow direct data sharing. However, the data analysed in this study would in principle be available to a researcher who applied to the data custodians and obtained the same approvals.
