# Supplementary material for: Hormone replacement therapy and cancer mortality in women with 17 site-specific cancers: a cohort study using linked medical records
Source: Br J Cancer. 2024 Jun 24;131(4):737–46. doi: 10.1038/s41416-024-02767-8 (PMC11333726; doi:10.1038/s41416-024-02767-8)

**Supplementary Information**

Supplementary tables and figures for the article “Hormone replacement therapy and cancer mortality in women with 17 site-specific cancers.”

Supplementary Table 1: International Classification of Diseases version 10 (ICD 10) codes of cancer diagnosis for cancer and cancer-specific mortality.

| Cancer Site | Cancer diagnosis codes^a^ | Cancer-specific mortality  codes |
| --- | --- | --- |
| Colorectal | C18-C20 | C18-C20, C26 |
| Oesophagus | C15 | C15, C26, C26 |
| Gastric | C16 | C15, C16, C26 |
| Liver | C22 | C22, C23, C24 |
| Pancreas | C25 | C25 |
|  |  |  |
| Lung | C33,C34 | C33,C34, C39 |
|  |  |  |
| Melanoma | C43 | C43 |
|  |  |  |
| Cervix | C53 | C53, C55, C57 |
| Ovary | C56 | C56, C57 |
| Uterus | C54 | C54, C55, C57 |
|  |  |  |
| Kidney | C64, C65 | C64, C65, C66, C68 |
| Bladder | C67 | C67, C68 |
|  |  |  |
| Brain | C71 | C71, C72 |
| Oral | C00-C14 | C00-C14 |
|  |  |  |
| Non-Hodgkin lymphoma | C82-C85 | C82-C85 |
| Myeloma | C90 | C90 |
| Leukaemia | C91-C95 | C91-C95 |

^a^Cancer was identified in the English dataset additionally using GP Read codes.

Supplementary Table 2: Pooled analyses of hormone replacement therapy use after diagnosis and all-cause mortality in England, Scotland and Wales.

|  | England^a^ | |  | | Scotland | |  | | Wales | |
| --- | --- | --- | --- | --- | --- | --- | --- | --- | --- | --- |
| Stage | HRT user | HRT non-user | | HRT user | | HRT non-user | | HRT user | | HRT non-user |
|  |  |  | |  | |  | |  | |  |
| Colorectal |  |  | |  | |  | |  | |  |
| 1 | 126 (16%) | 2258 (16%) | | 78 (24%) | | 1614 (19%) | | 35 (13%) | | 702 (13%) |
| 2 | 247 (31%) | 4308 (30%) | | 92 (28%) | | 2361 (27%) | | 55 (20%) | | 1279 (24%) |
| 3 | 238 (30%) | 4409 (31%) | | 84 (25%) | | 2216 (25%) | | 74 (27%) | | 1584 (29%) |
| 4 | 75 (9%) | 1760 (12%) | | 30 (9%) | | 1182 (14%) | | 14 (5%) | | 459 (9%) |
| Missing | 110 (14%) | 1504 (11%) | | 47 (14%) | | 1349 (15%) | | 101 (36%) | | 1373 (25%) |
|  |  |  | |  | |  | |  | |  |
| Lung |  |  | |  | |  | |  | |  |
| 1 | 65 (18%) | 1480 (20%) | | 75 (34%) | | 2688 (30%) | | 26 (15%) | | 610 (16%) |
| 2 | 29 (8%) | 618 (8%) | | 17 (8%) | | 686 (8%) | | 7 (4%) | | 291 (7%) |
| 3 | 62 (17%) | 1607 (21%) | | 47 (22%) | | 2302 (25%) | | 16 (9%) | | 751 (19%) |
| 4 | 83 (22%) | 2330 (31%) | | 68 (31%) | | 2805 (31%) | | 20 (11%) | | 829 (21%) |
| Missing | 130 (35%) | 1534 (20%) | | 11 (5%) | | 596 (7%) | | 107 (61%) | | 1436 (37%) |
|  |  |  | |  | |  | |  | |  |
| Melanoma |  |  | |  | |  | |  | |  |
| 1 | 315 (85%) | 2648 (78%) | | 271 (74%) | | 2052 (59%) | | 147 (55%) | | 1125 (53%) |
| 2 | 42 (11%) | 522 (15%) | | 55 (15%) | | 686 (20%) | | 30 (11%) | | 349 (17%) |
| 3 | 8 (2%) | 182 (5%) | | 26 (7%) | | 387 (11%) | | 0-6^b^ (%) | | 72 (3%) |
| 4 | 3 (1%) | 45 (1%) | | 6 (2%) | | 225 (6%) | | 0-6^b^ (%) | | 39 (2%) |
| Missing | 4 (1%) | 14 (0%) | | 9 (2%) | | 138 (4%) | | 84 (31%) | | 523 (25%) |
|  |  |  | |  | |  | |  | |  |
| Cervical |  |  | |  | |  | |  | |  |
| 1 | 92 (23%) | 293 (20%) | | 237 (70%) | | 484 (38%) | | 113 (55%) | | 244 (32%) |
| 2 | 31 (8%) | 156 (11%) | | 78 (23%) | | 398 (31%) | | 31 (15%) | | 128 (17%) |
| 3 | 15 (4%) | 127 (9%) | | 9 (3%) | | 150 (12%) | | 0-12^b^ (%) | | 78 (10%) |
| 4 | 7 (2%) | 113 (8%) | | 8 (2%) | | 152 (12%) | | 0-12^b^ (%) | | 66 (9%) |
| Missing | 253 (64%) | 772 (53%) | | 8 (2%) | | 106 (8%) | | 51 (25%) | | 255 (33%) |
|  |  |  | |  | |  | |  | |  |
| Ovarian |  |  | |  | |  | |  | |  |
| 1 | 287 (50%) | 1470 (29%) | | 155 (75%) | | 1126 (35%) | | 83 (31%) | | 455 (19%) |
| 2 | 18 (3%) | 313 (6%) | | 8 (4%) | | 243 (8%) | | 13 (5%) | | 144 (6%) |
| 3 | 119 (21%) | 1600 (32%) | | 15 (7%) | | 1105 (35%) | | 0-16^b^ (%) | | 436 (19%) |
| 4 | 36 (6%) | 740 (15%) | | 11 (5%) | | 512 (16%) | | 0-16^b^ (%) | | 154 (7%) |
| Missing | 116 (20%) | 890 (18%) | | 19 (9%) | | 213 (7%) | | 152 (58%) | | 1163 (49%) |

^a^Restricted to individuals with linked cancer registry data. ^b^Range shown to maintain statistical disclosure control.

Supplementary Table 3: Sensitivity analyses for the association between hormone replacement therapy use after diagnosis and cancer-specific mortality adjusting for BMI in England and Wales.

| Analysis | Events^a^ | Person-years^a^ | Unadjusted^a^ HR (95% CI) | Adjusted for main model + BMI^b^  HR (95% CI) | Main analysis adjusted^c^ HR (95% CI) |
| --- | --- | --- | --- | --- | --- |
| Colorectal | 4563 | 105566 | 0.78 (0.67, 0.91) | 0.78 (0.66, 0.91) | 0.79 (0.70, 0.90) |
| Oesophagus | 1249 | 5426 | 0.81 (0.59, 1.10) | 0.88 (0.64, 1.21) | 0.93 (0.72, 1.19) |
| Gastric | 888 | 6914 | 0.49 (0.17, 1.39) | 0.58 (0.24, 1.37) | 0.81 (0.47, 1.42) |
| Liver | 617 | 2159 | 1.19 (0.75, 1.88) | 1.43 (0.87, 2.35) | 1.11 (0.74, 1.66) |
| Pancreas | 1730 | 4401 | 0.79 (0.58, 1.08) | 0.86 (0.62, 1.17) | 0.84 (0.65, 1.09) |
|  |  |  |  |  |  |
| Lung | 8469 | 30428 | 1.04 (0.93, 1.15) | 1.01 (0.91, 1.13) | 0.98 (0.90, 1.07) |
|  |  |  |  |  |  |
| Melanoma | 673 | 67420 | 0.78 (0.45, 1.35) | 0.98 (0.48, 2.00) | 0.77 (0.58, 1.02) |
|  |  |  |  |  |  |
| Cervix | 552 | 15571 | 0.50 (0.38, 0.67) | 0.82 (0.60, 1.11) | 0.82 (0.66, 1.02) |
| Ovary | 3558 | 41889 | 0.43 (0.31, 0.60) | 0.74 (0.64, 0.86) | 0.60 (0.39, 0.93) |
| Uterus | 1324 | 80104 | 0.27 (0.15, 0.47) | 0.41 (0.23, 0.72) | 0.43 (0.27, 0.67) |
|  |  |  |  |  |  |
| Kidney | 880 | 21365 | 0.46 (0.31, 0.69) | 0.55 (0.33, 0.91) | 0.55 (0.40, 0.76) |
|  |  |  |  |  |  |
| Bladder | 759 | 29539 | 0.69 (0.29, 1.64) | 0.96 (0.39, 2.35) | 0.85 (0.49, 1.48) |
|  |  |  |  |  |  |
| Brain | 923 | 3883 | 0.87 (0.63, 1.20) | 0.86 (0.61, 1.20) | 1.01 (0.79, 1.29) |
| Oral | 633 | 18298 | 0.60 (0.34, 1.06) | 0.59 (0.38, 0.91) | 0.58 (0.42, 0.80) |
|  |  |  |  |  |  |
| Non-Hodgkin lymphoma | 1192 | 37678 | 0.51 (0.23, 1.13) | 0.74 (0.44, 1.25) | 0.77 (0.60, 0.99) |
| Myeloma | 924 | 12118 | 0.62 (0.19, 1.97) | 0.58 (0.13, 2.54) | 0.88 (0.63, 1.23) |
| Leukaemia | 861 | 23184 | 0.79 (0.59, 1.06) | 1.00 (0.70, 1.44) | 0.79 (0.61, 1.03) |

^a^Events, person years and unadjusted hazard ratio restricted to individuals with available BMI in England and Wales. ^b^Adjusted model contains age, year of diagnosis, deprivation, cancer treatment (surgery, radiotherapy, chemotherapy), Charlson comorbidities (before diagnosis), anaemia(before diagnosis), medication use (before diagnosis: statin, aspirin, metformin and oral contraceptive) and hysterectomy/oophorectomy (before or at diagnosis) and BMI (continuous). ^c^Adjusted HR from main analysis in England, Scotland and Wales. Model contains age, year of diagnosis, deprivation, cancer treatment (surgery, radiotherapy, chemotherapy), Charlson comorbidities (before diagnosis), anaemia(before diagnosis), medication use (before diagnosis: statin, aspirin, metformin and oral contraceptive) and hysterectomy/oophorectomy (before or at diagnosis).

Supplementary Table 4: Additional pooled sensitivity analyses for the association between hormone replacement therapy use after diagnosis and cancer-specific mortality in England, Scotland and Wales.

| Analysis | Events | Person-years | Unadjusted HR (95% CI) | Adjusted^a^ HR (95% CI) |
| --- | --- | --- | --- | --- |
| Colorectal |  |  |  |  |
| Main analysis | 8481 | 178687 | 0.80 (0.70, 0.91) | 0.79 (0.70, 0.90) |
| Including vaginal oestrogen therapy | 8481 | 178687 | 0.77 (0.67, 0.89) | 0.79 (0.70, 0.90) |
| Including age 18 to 79 years | 8662 | 182968 | 0.80 (0.72, 0.90) | 0.79 (0.71, 0.89) |
| Adjusted for stage and smoking (CC)^b^ | 4289 | 96259 | 0.89 (0.76, 1.05) | 0.84 (0.67, 1.06) |
| Outcome death from CVD | 1411 | 178687 | 0.41 (0.30, 0.55) | 0.87 (0.64, 1.19) |
| Restricted to stage 1 and 2 | 1326 | 84712 | 0.74 (0.52, 1.05) | 0.77 (0.57, 1.05) |
|  |  |  |  |  |
| Lung |  |  |  |  |
| Main analysis | 16504 | 58215 | 0.97 (0.87, 1.08) | 0.98 (0.90, 1.07) |
| Including vaginal oestrogen therapy | 16504 | 58215 | 0.87 (0.76, 0.99) | 0.89 (0.78, 1.02) |
| Including age 18 to 79 years | 16565 | 58969 | 0.98 (0.88, 1.08) | 0.99 (0.91, 1.08) |
| Adjusted for stage and smoking (CC)^b^ | 5972 | 21513 | 1.09 (0.95, 1.25) | 1.19 (1.01, 1.40) |
| Outcome death from CVD | 1156 | 58215 | 0.54 (0.38, 0.78) | 0.78 (0.54, 1.12) |
| Restricted to stage 1 and 2 | 2258 | 22475 | 0.88 (0.54, 1.43) | 1.25 (0.95, 1.66) |
|  |  |  |  |  |
| Melanoma |  |  |  |  |
| Main analysis | 1152 | 108702 | 0.68 (0.50, 0.93) | 0.77 (0.58, 1.02) |
| Including vaginal oestrogen therapy | 1152 | 108702 | 0.66 (0.52, 0.82) | 0.70 (0.56, 0.88) |
| Including age 18 to 79 years | 1249 | 131102 | 0.73 (0.54, 0.99) | 0.77 (0.58, 1.02) |
| Adjusted for stage and smoking (CC) ^b^ | 299 | 26646 | 0.66 (0.23, 1.90) | 0.85 (0.49, 1.47) |
| Outcome death from CVD | 375 | 108702 | 0.50 (0.19, 1.33) | 1.40 (0.40, 4.95) |
| Restricted to stage 1 and 2 | 292 | 46770 | 0.76 (0.47, 1.23) | 0.95 (0.57, 1.56) |
|  |  |  |  |  |
| Cervix |  |  |  |  |
| Main analysis | 1213 | 30649 | 0.46 (0.34, 0.62) | 0.82 (0.66, 1.02) |
| Including vaginal oestrogen therapy | 1213 | 30649 | 0.50 (0.40, 0.61) | 0.81 (0.66, 0.98) |
| Including age 18 to 79 years | 1542 | 53812 | 0.86 (0.74, 0.99) | 1.10 (0.94, 1.28) |
| Adjusted for stage and smoking (CC)^b^ | 504 | 12448 | 0.56 (0.42, 0.74) | 1.10 (0.70, 1.71) |
| Outcome death from CVD^c^ | 134 | 22719 | 0.28 (0.15, 0.50) | 0.63 (0.33, 1.22) |
| Restricted to stage 1 and 2 | 244 | 11778 | 0.62 (0.36, 1.05) | 1.06 (0.70, 1.61) |
|  |  |  |  |  |
| Ovary |  |  |  |  |
| Main analysis | 6056 | 68868 | 0.39 (0.27, 0.57) | 0.60 (0.39, 0.93) |
| Including vaginal oestrogen therapy | 6056 | 68868 | 0.47 (0.39, 0.57) | 0.68 (0.52, 0.88) |
| Including age 18 to 79 years | 6181 | 77246 | 0.36 (0.25, 0.53) | 0.64 (0.43, 0.94) |
| Adjusted for stage and smoking (CC)^b^ | 2636 | 27137 | 0.44 (0.28, 0.70) | 0.83 (0.68, 1.02) |
| Outcome death from CVD^c^ | 405 | 55183 | 0.24 (0.15, 0.38) | 0.55 (0.34, 0.89) |
| Restricted to stage 1 and 2 | 587 | 24537 | 0.45 (0.26, 0.79) | 0.66 (0.41, 1.06) |
|  |  |  |  |  |

^a^Adjusted model contains age, year of diagnosis, deprivation, cancer treatment (surgery, radiotherapy, chemotherapy), Charlson comorbidities (before diagnosis), anaemia(before diagnosis), medication use (before diagnosis: statin, aspirin, metformin and oral contraceptive) and hysterectomy/oophorectomy (before or at diagnosis).

^b^Restricted to individuals with available stage and smoking data in Wales and England. Adjusted model contains all terms in ^a^ along with stage and smoking status

^c^Excluding Scotland due to small numbers.

Supplementary Table 5: Pooled analyses of hormone replacement therapy use by type and cancer-specific mortality after diagnosis in England, Scotland and Wales.

| Analysis | Cancer- deaths | Person-years | Unadjusted HR (95% CI) | Adjusted^b^ HR (95% CI) |
| --- | --- | --- | --- | --- |
| Colorectal |  |  |  |  |
| HRT non-use | 8192 | 167432 | 1.00 (ref. cat.) | 1.00 (ref. cat.) |
| Oestrogen plus progestogen**^a^** | 131 | 5311 | 0.78 (0.56, 1.10) | 0.78 (0.56, 1.08) |
| Oestrogen alone**^a^** | 128 | 4823 | 0.81 (0.68, 0.96) | 0.80 (0.64, 0.98) |
| Tibolone**^a^** | 25-30^c^ | 1124 | 0.84 (0.59, 1.20) | 0.84 (0.59, 1.20) |
|  |  |  |  |  |
| Lung |  |  |  |  |
| HRT non-use | 15931 | 55229 | 1.00 (ref. cat.) | 1.00 (ref. cat.) |
| Oestrogen plus progestogen**^a^** | 270 | 1496 | 0.97 (0.86, 1.09) | 0.95 (0.84, 1.08) |
| Oestrogen alone**^a^** | 245 | 1223 | 0.94 (0.79, 1.12) | 0.95 (0.76, 1.18) |
| Tibolone**^a^** | 58 | 267 | 1.14 (0.88, 1.47) | 1.07 (0.82, 1.38) |
|  |  |  |  |  |
| Melanoma |  |  |  |  |
| HRT non-use | 1078 | 95824 | 1.00 (ref. cat.) | 1.00 (ref. cat.) |
| Oestrogen plus progestogen**^a^** | 39 | 7010 | 0.69 (0.49, 0.97) | 0.77 (0.53, 1.13) |
| Oestrogen alone**^a^** | 22-32 | 5169 | 0.61 (0.38, 0.99) | 0.72 (0.48, 1.08) |
| Tibolone**^a^** | 6-16^c^ | 648 | 1.31 (0.65, 2.64) | 1.35 (0.67, 2.71) |
|  |  |  |  |  |
| Cervix |  |  |  |  |
| HRT non-use | 1102 | 22788 | 1.00 (ref. cat.) | 1.00 (ref. cat.) |
| Oestrogen plus progestogen**^a^** | 54 | 2021 | 0.83 (0.63, 1.09) | 0.94 (0.71, 1.26) |
| Oestrogen alone**^a^** | 32-42^c^ | 5133 | 0.21 (0.09, 0.51) | 0.65 (0.47, 0.91) |
| Tibolone**^a^** | 15-25^c^ | 707 | 1.98 (0.28, 14.20) | 2.65 (0.51, 13.85) |
|  |  |  |  |  |
| Ovary |  |  |  |  |
| HRT non-use | 5735 | 58518 | 1.00 (ref. cat.) | 1.00 (ref. cat.) |
| Oestrogen plus progestogen**^a^** | 78-88 | 1323 | 0.75 (0.43, 1.28) | 0.86 (0.46, 1.60) |
| Oestrogen alone**^a^** | 208 | 8464 | 0.31 (0.21, 0.46) | 0.55 (0.38, 0.79) |
| Tibolone**^a^** | 30-35^c^ | 564 | 0.73 (0.52, 1.05) | 0.93 (0.58, 1.49) |

^a^HRT type categorised into users of oestrogen plus progestogen (with or without oestrogen alone or tibolone users), users of oestrogen alone (with or without tibolone use) and users of tibolone.

^b^Adjusted model contains age, year of diagnosis, deprivation, cancer treatment (surgery, radiotherapy, chemotherapy), Charlson comorbidities (before diagnosis), anaemia (before diagnosis), medication use (before diagnosis: statin, aspirin, metformin and oral contraceptive) and hysterectomy/oophorectomy (before or at diagnosis).

^c^Range shown to maintain statistical disclosure control.

Supplementary Table 6: Pooled analyses of hormone replacement therapy use after diagnosis and all-cause mortality in England, Scotland and Wales.

| **Cancer site** | **HRT user** | |  | | **HRT non-user** | | **Unadjusted HR (95% CI)** | **P^b^** | **Adjusted^a^ HR (95% CI)** | **P^c^** |
| --- | --- | --- | --- | --- | --- | --- | --- | --- | --- | --- |
|  | All deaths | Person-years | | All deaths | | Person-years |  |  |  |  |
| Colorectal | 469 | 11256 | | 12783 | | 167432 | 0.66 (0.59, 0.75) | <0.001 | 0.78 (0.71, 0.86) | <0.001 |
| Oesophagus | 81 | 568 | | 2736 | | 8818 | 0.78 (0.63, 0.98) | 0.031 | 0.90 (0.72, 1.14) | 0.39 |
| Gastric | 68 | 803 | | 2411 | | 10758 | 0.61 (0.41, 0.92) | 0.017 | 0.74 (0.45, 1.22) | 0.237 |
| Liver | 28-33^d^ | 103 | | 1337 | | 3612 | 0.99 (0.70, 1.41) | 0.965 | 0.97 (0.60, 1.57) | 0.896 |
| Pancreas | 75 | 337 | | 3141 | | 6317 | 0.76 (0.60, 0.95) | 0.019 | 0.85 (0.67, 1.08) | 0.183 |
|  |  |  | |  | |  |  |  |  |  |
| Lung | 679 | 2986 | | 18822 | | 55229 | 0.88 (0.75, 1.04) | 0.131 | 0.94 (0.83, 1.07) | 0.349 |
|  |  |  | |  | |  |  |  |  |  |
| Melanoma | 151 | 12877 | | 2171 | | 95824 | 0.54 (0.46, 0.64) | <0.001 | 0.82 (0.61, 1.11) | 0.195 |
|  |  |  | |  | |  |  |  |  |  |
| Cervix | 159 | 7862 | | 1642 | | 22788 | 0.37 (0.28, 0.49) | <0.001 | 0.71 (0.60, 0.85) | <0.001 |
| Ovary | 399 | 10350 | | 7249 | | 58518 | 0.35 (0.25, 0.49) | <0.001 | 0.57 (0.40, 0.81) | 0.002 |
| Uterus | 98 | 5837 | | 5411 | | 124788 | 0.44 (0.36, 0.54) | <0.001 | 0.71 (0.54, 0.93) | 0.012 |
|  |  |  | |  | |  |  |  |  |  |
| Kidney | 78 | 2848 | | 2434 | | 34047 | 0.46 (0.37, 0.58) | <0.001 | 0.65 (0.50, 0.85) | 0.002 |
| Bladder | 104 | 3100 | | 2701 | | 38680 | 0.58 (0.39, 0.85) | 0.005 | 0.77 (0.49, 1.20) | 0.242 |
|  |  |  | |  | |  |  |  |  |  |
| Brain | 85 | 473 | | 1857 | | 5621 | 0.95 (0.76, 1.18) | 0.618 | 1.01 (0.81, 1.27) | 0.903 |
| Oral | 93 | 2614 | | 2349 | | 28711 | 0.54 (0.44, 0.66) | <0.001 | 0.60 (0.48, 0.75) | <0.001 |
|  |  |  | |  | |  |  |  |  |  |
| Non-Hodgkin lymphoma | 159 | 5293 | | 3483 | | 56960 | 0.49 (0.33, 0.72) | <0.001 | 0.78 (0.58, 1.04) | 0.092 |
| Myeloma | 116 | 1322 | | 2200 | | 18029 | 0.73 (0.59, 0.91) | 0.005 | 0.91 (0.75, 1.10) | 0.33 |
| Leukaemia | 125 | 3011 | | 2460 | | 32361 | 0.59 (0.42, 0.84) | 0.003 | 0.86 (0.68, 1.08) | 0.182 |

**^a^**Adjusted model contains age, year of diagnosis, deprivation, cancer treatment (surgery, radiotherapy, chemotherapy), Charlson comorbidities (before diagnosis), anaemia (before diagnosis), medication use (before diagnosis: statin, aspirin, metformin and oral contraceptive) and hysterectomy/oophorectomy (before or at diagnosis). ^b^P-value from unadjusted Cox regression model. ^c^P-value from adjusted Cox regression model. ^d^Range shown to maintain statistical disclosure control.

Supplementary Figure 1: Adjusted hazard ratios for the association between hormone replacement therapy use before diagnosis and cancer-specific mortality mortality in England (♦), Scotland (●), Wales (▲) and pooled (■), by site.


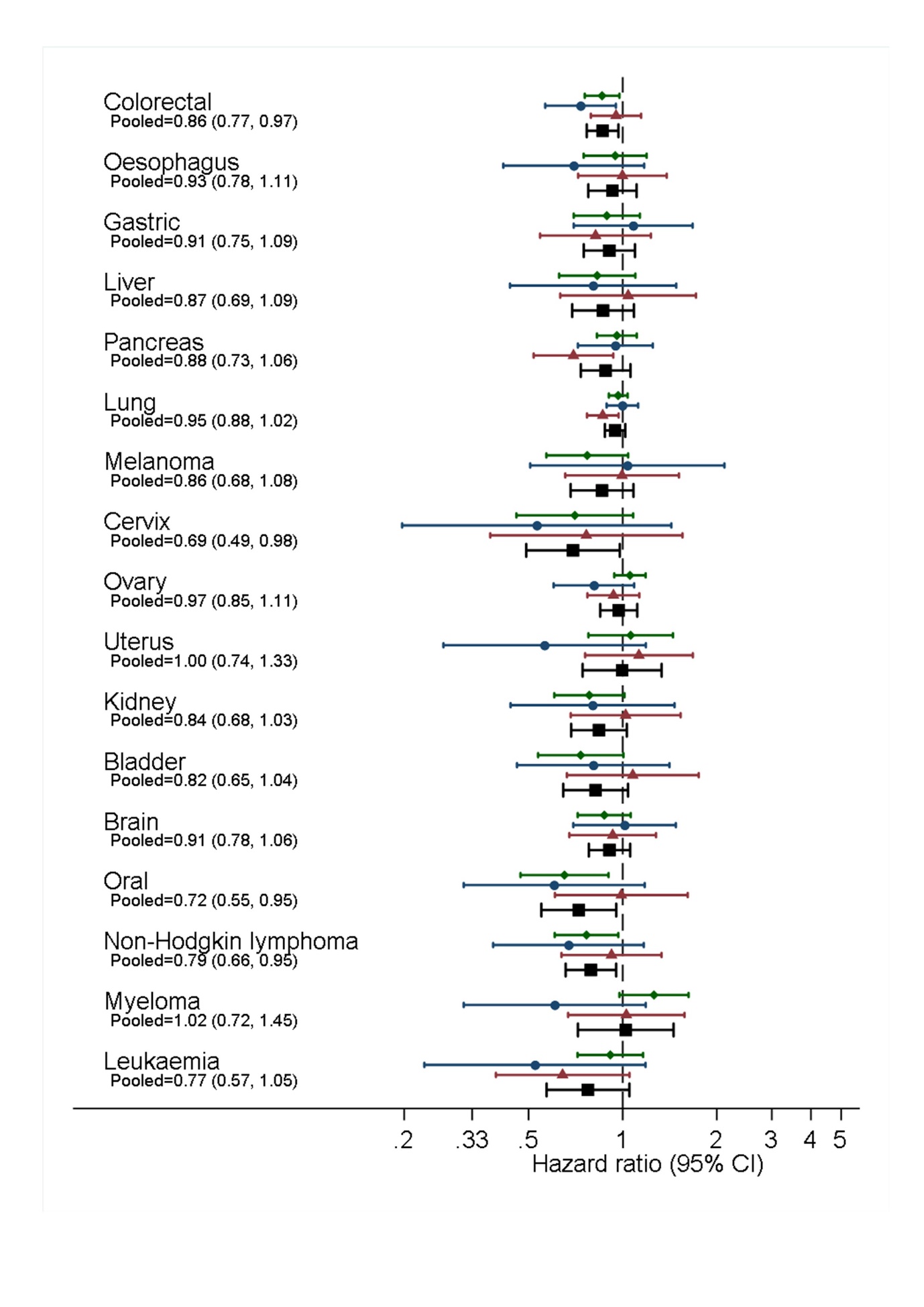

Supplement: Supplementary file 1 — Supplementary Information [file 41416_2024_2767_MOESM1_ESM.docx]
